# The Pro-Inflammatory Cytokine, Interleukin-6, Enhances the Polarization of Alternatively Activated Macrophages

**DOI:** 10.1371/journal.pone.0094188

**Published:** 2014-04-15

**Authors:** Maria Ruweka Fernando, Jose Luis Reyes, Jordan Iannuzzi, Gabriella Leung, Derek Mark McKay

**Affiliations:** Gastrointestinal Research Group, Inflammation Research Network, Department of Physiology and Pharmacology, The Calvin Phoebe and Joan Snyder Institute for Chronic Diseases, University of Calgary, Calgary, Alberta, Canada; McMaster University, Canada

## Abstract

Macrophages are important innate immune cells that are associated with two distinct phenotypes: a pro-inflammatory (or classically activated) subset with prototypic macrophage functions such as inflammatory cytokine production and bactericidal activity, and an anti-inflammatory (or alternatively activated (AAM)) subset linked with wound healing and tissue repair processes. In this study, we examined the effect of interlukein-6 on human and murine macrophage polarization. The results indicate that despite being commonly associated with pro-inflammatory functions and being implicated in the pathogenesis/pathophysiology of numerous inflammatory diseases, interleukin-6 can enhance the polarization of AAMs, based on increased expression of hallmark markers: arginase-1, Ym1 and CD206; this effect required the AAM differentiating cytokines, IL-4 and IL-13. Co-treatment of AAMs with IL-6 resulted in spontaneous release of IL-10, suppressed LPS-induced nitric oxide production and inhibited cytokine production by activated CD4^+^ T cells – immunoregulatory features not observed in the ‘parent’ IL-4+IL-13-induced AAM. The effect of IL-6 required signal transducer and activator of transcription (STAT)-3, was partially dependent on up-regulation of the IL4Rα chain, and was independent of autocrine IL-10. In the presence of IFNγ, IL-6 promoted the production of IL-1β and TNFα suggesting that this cytokine can enhance the phenotype to which a macrophage has committed. This finding may explain the pleiotrophic nature of IL-6, where it is associated with the perpetuation and enhancement of disease in inflammatory situations, but is also necessary for resolution of inflammation and adequate wound healing to occur in others. Thus, the potential benefit of IL-6 in promoting an AAM, with its’ anti-inflammatory and wound healing ability, may need to be considered in immunotherapies aimed at *in vivo* modulation or inhibition of IL-6.

## Introduction

Macrophages are a major component of the innate immune system, and function as one of the earliest lines of defence against invading pathogens [1]. Additionally, they are critical in the maintenance of tissue homeostasis and the turnover of tissue and organ systems. Upon their discovery by Metchnikoff and until recently, macrophages were primarily associated with pro-inflammatory and bactericidal functions. However, it is now known that different subpopulations of macrophages exist, carrying out distinct, but at times overlapping, functions [1]. These subpopulations can be generally categorized as classically activated macrophages (CAMs or M1 macrophages), which are the prototypic pro-inflammatory macrophage subset induced by exposure to interferon-γ (IFNγ) and/or lipopolysaccharide (LPS), or alternatively activated macrophages (AAMs or M2 macrophages) [1], [2]. Since their initial discovery as macrophages induced by IL-4 and expressing increased levels of the mannose receptor (MRC1/CD206), various other anti-inflammatory macrophage subsets have been discovered. In one classification, AAMs differentiated by IL-4 and/or IL-13 are M2a macrophages, macrophages polarized by immune complexes and secreting high levels of IL-10 are M2b macrophages and those cells ‘deactivated’ by exposure to IL-10 are referred to as M2c macrophages [3]. Others have proposed a spectrum of macrophage activation, with CAMs, AAMs (associated with wound healing) and regulatory macrophages (involved in immune responses but not wound healing) making up the three primary subdivisions with various other identified populations in-between [1]. In addition to these subtypes, there exists (among others) tumor associated macrophages (TAMs) and myeloid-derived suppressor cells (MDSCs), which are M2-like cells [4]. In this study, we examined IL-4+IL-13-induced or M2a macrophages (hereafter referred to as AAMs), which in mice are characterized by increased expression of arginase 1 (Arg1), Ym1 (a chitinase-like molecule) and RELMα (resistin-like molecule α). In humans, other markers, such as PPARγ and CD206 can be used for identification of AAMs [5].

Macrophages are phenotypically plastic cells that are highly influenced by the microenvironment in which they reside, and exposure to differentiating cytokines such as IL-4/IL-13 and IFNγ does not induce terminal differentiation [6]–[8]. Several studies have shown that the AAM phenotype can be enhanced by various cytokines, such as IL-33, as well as through interactions with fibroblasts and regulatory T cells [9]–[11]. Alternatively, the AAM phenotype can be ‘broken’ or inhibited in a variety of ways, such as exposure to IFNγ/TNFα [12]. Given these data, we were primarily interested in investigating the role of interleukin (IL)–6 on AAM function. IL-6 is often considered a pro-inflammatory cytokine found in higher levels in a number of diseases, including IBD [13], and its inhibition in rheumatoid arthritis has proved to be a beneficial [14]. A key function of IL-6 is its role as a regulator of the balance between regulatory T cells and Th17 cells – IL-6 can inhibit the formation of Tregs and promote the formation of Th17 cells [15]. In light of the similarities between Tregs and AAMs as immunoregulatory cells, and Th17 cells and CAMs, we were interested in determining whether IL-6 had a similar regulatory role in mediating the CAM-AAM balance.

Using an *in vitro* approach we show that contrary to our expectation, IL-6 can potently enhance and sustain AAMs, conferring additional immunosuppressive functions. This illustrates the Janus–nature of IL-6 which appears to have the ability to enforce the phenotype that the micro-environment commits a macrophage to, whether AAM or CAM.

## Materials and Methods

### Mice

All animal experiments complied with Canadian and Institutional guidelines for animal welfare (experiments were approved by the Health Science Animal Care Committee (HSACC) at the University of Calgary. Male BALB/c and C57/Bl6 mice were purchased from Charles River (Quebec, CA). IL-10^-/-^ mice were purchased from Jackson Labs (Sacramento, CA, USA).

### Murine Bone Macrophage Culture

Bone-marrow was isolated from murine femurs and cultured in RPMI1640 supplemented with 20% fetal bovine serum (FBS), 1.2% GlutaMAX, 2.4% penicillin-streptomycin (all from Invitrogen Canada Inc., Burlington, ON) and 20 ng/mL mouse M-CSF (R&D Systems Inc., Minneapolis, MN) for 7 days as described for bone-marrow derived macrophage (BMDM) differentiation [9]. BMDM were stimulated for 48 h with IL-4+IL-13 (both at 20 ng/mL [9], Cedarlane Laboratories, Burlington, ON) to induce AAMs or IFNγ (10 ng/mL, Cedarlane) to induce CAMs±IL-6 (10 ng/mL, Cedarlane). For some experiments, macrophages were also incubated with IL-4+IL-13 plus IL-10, IL-11, leukemia inhibitory factor (LIF), IFNγ or TNFα (all 10 ng/mL, Cedarlane). To assess nitric oxide and cytokine production, cells were rinsed with PBS and challenged with LPS (1 µg/mL) for an additional 24 h following cytokine stimulation. Macrophages were assessed for cytokine production (IL-10, TNFα, IL-1β and CCL17) via ELISA before and after LPS stimulation, following the manufacturers instructiosn (R&D Systems).

### Human Macrophage Culture

Briefly, blood was collected from healthy consented volunteers, mixed with PBS + 2% FBS and overlaid on 37°C Ficoll-Plaque PLUS (GE Healthcare, Bio-Sciences AB, Uppsala, Sweden). Cells were centrifuged at 400x*g* for 30 min without brakes and the buffy coat (containing monocytes) was collected and washed twice in PBS. Cells were then seeded in serum-free medium for 2 h for monocyte adherence, rinsed twice with PBS to remove non-adherent cells and re-fed with serum-containing medium. Monocytes were cultured for 7 days and then differentiated into AAMs with IL-4+IL-13±IL-6 treatment (all cytokines at 10 ng/mL).

### Arginase Assay

Arginase activity in murine macrophages was assessed by measuring urea production, a by-product of the arginase reaction, as previously described. Arginase activity is expressed as units per 10^6^ cells, where 1 unit equals the amount of enzyme needed to hydrolyse 1 µM of arginine/min. Arginase activity was determined based on a standard curve of known urea concentrations [16].

### Nitric Oxide (Griess) Assay

Nitric oxide production was determined by measuring nitrite, a stable break-down product of nitric oxide metabolism, in cell supernatants. Supernatants were combined with an equal volume of 2% sulphanilamide (Sigma-Aldrich Canada, Ltd., Oakville, ON) and 0.1% N-1-naphthylethylenediamine dihydrochloride (Sigma-Aldrich) to convert nitrite into a magenta colored azo-compound with a measurable absorbance at 540 nm. Nitrite levels were determined based on a standard curve of known sodium nitrite concentrations.

### Immunoblotting

Briefly, cells were lysed in modified RIPA buffer (50 mM Tris-HCl, 150 mM NaCl, 1% NP-40, 0.5% sodium deoxycholate and 0.1% SDS) supplemented with protease inhibitor cocktail (Promega, Madison, WI) and protein concentrations determined via the Bradford assay (Bio-Rad Laboratories Canada, Mississauga, ON, Canada). Samples (10–20 µg) were boiled for 10 min with 4x Laemelli buffer, run by SDS-PAGE (4% stacking, 8% separating) and transferred to a nitrocellulose membrane. Membranes were blocked for 1 h at room temperature in 5% skim milk in 0.1% TBS-T (Tris buffered saline, 0.1% Tween 20) and then incubated overnight with rabbit polyclonal antibodies to arginase-1 (1∶1000, Santa Cruz Biotechnology Inc, Santa Cruz, CA), Ym1 (1∶1000, Stem Cell Technologies, Vancouver, BC), signal transducer and activator of transcription (STAT)-3 (1∶1000, Cell Signalling Technology, Danvers, MA), phospho-STAT3 (1∶1000, Cell Signalling Technology), phospho-STAT6 (1∶1000, Cell Signalling Technology), a mouse monoclonal antibody to iNOS (1∶750, BD Biosciences, Mississauga, ON) and a goat polyclonal antibody to β-actin (1∶1000, Santa Cruz). Membranes were then incubated with appropriate anti-goat, anti-rabbit or anti-mouse secondary antibodies (all at 1∶2000, Santa Cruz) for 1 h at room temperature. Membranes were washed again, exposed to Western Lightning Plus Enhanced Chemiluminescence Solution (PerkinElmer, Woodbridge, ON) for 1 min, exposed to X-Omat Blue film (PerkinElmer) for 5 sec to 10 min and developed using an automatic film developer.

### qPCR

RNA was isolated using TRIzol (Invitrogen) and quantified using a NanoDrop (Thermo Scientific), as previously described [17]. Briefly, 1 ng of isolated RNA was used to generate cDNA via iScript RT kit (Bio-Rad) in a MyCycler thermocycler (Bio-Rad). cDNA was added to 300 nM gene-specific primers (primer sequences are available in Table 1) and 1x SYBR green reaction mix (Bio-Rad). Changes in gene expression were assessed using the Mastercycler Real Time RT PCR Thermocycler (Eppendorf Canada, Mississauga, ON) as described [17] and data analyzed using the 2CT method using 18s as a house-keeping gene and normalized to expression in untreated controls [18].

**Table 1:** Sequences of PCR primers used in this study.

Primer	Forward	Reverse
Murine		
Arg1	AACACTCCCCTGACAACCAG	CCAGCAGGTAGCTGAAGGTC
Ym1	TGGAGGATGGAAGTTTGGAC	AATGATTCCTGCTCCTGTGG
RELMα	CCCTTCTCATCTGCATCTCC	CAGTAGCAGTCATCCCAGCA
IL-4Rα	CCTCACACTCCACACCAATG	AGCCTGGGTTCCTTGTAGGT
18s	CGCGGTTCTATTTTGTTGGT	AGTCGGCATCGTTTATGGTC
Human		
CD206	GGCGGTGACCTCACAAGTAT	ACGAAGCCATTTGGTAAACG
CCL18	CCCCAAGCCAGGTGTCATCCTC	GGGCCATTGCCCTGGCTCAG
18s	ATACATGCCGACGGGCGCTG	AGGGGCTGACCGGGTTGGTT

### Transfection of Macrophages with small interfering (si) RNA

Macrophages were transfected using a slightly modified protocol [19]. Briefly, 80 µM STAT3 siRNA (Santa Cruz) or Silencer Select Negative Control (Invitrogen) in RNAiMax+OptiMEM (Invitrogen) was added to 1×10^6^ macrophages in antibiotic-free RPMI1640 medium. Cells were incubated overnight at 37°C, rinsed the next day to remove dead cells and treated with IL-4+IL-13±IL-6 for 48 h. Cells were collected for analysis via western blot or arginase assay, or stimulated with LPS to assess nitric oxide production.

### Macrophage- T cell co-culture

Macrophage-T cell co-culture experiments were based on a slightly modified published protocols [20]. Splenocytes were harvested from naïve BALB/c mice and CD4^+^ T cells were purified using a magnetic enrichment kit (EasySep, Stem Cell Technologies) and 5×10^4^, 1×10^5^ or 2×10^5^ cells incubated with anti-CD28 (0.5 µg/mL) in 24-well plates that had been coated with 0.5 µg/mL anti-CD3 antibody (BD Bioscience) for 24 h at 37°C. Then, 5×10^4^ AAMs or AAMs(IL-6) were added. Supernatants were collected 96 h later and analyzed by ELISA for IL-2, IL-4 and IFNγ production (R&D Systems).

### Data Presentation and Statistical Analysis

Unless stated otherwise AAM and CAM denotes macrophages treated with IL-4+IL-13 or IFNγ, respectively, ± IL-6 (i.e. AAM(IL-6)) (or other cytokine) being added simultaneously for a 48 h incubation. Data are presented as mean±standard error of the mean (SEM), and were analyzed using GraphPad Prism 5 software (GraphPad Software, La Jolla, CA) by one-way ANOVA followed by post-hoc analysis using Tukey’s test with p<0.05 accepted as a level of statistical significance.

## Results

### Recombinant IL-6 enhances the development of an AAM phenotype

**Figure 1:**
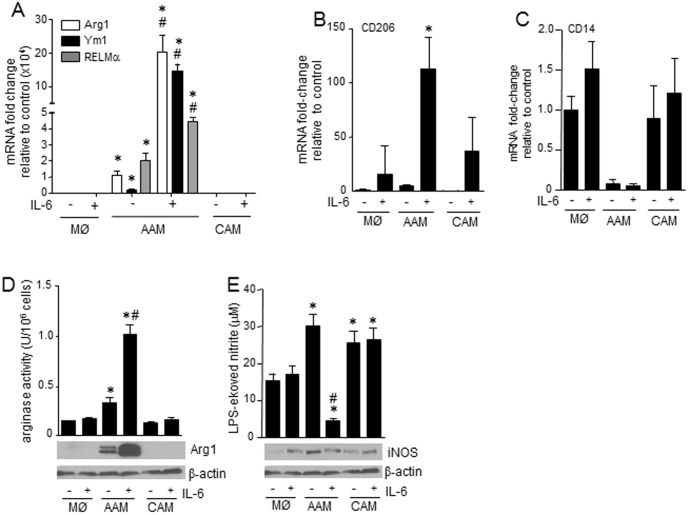
IL-6 enhances the polarization of murine bone-marrow derived AAMs. (A–C) Recombinant IL-6 (10 ng/mL, 48 h) enhance mRNA expression of markers of AAMs (Arg1, Ym1, RELMα and CD206) while not affecting the reduced expression of CD14 mRNA in AAMs (IL-4+IL-13, 20 ng/mL, 48 h) (n = 3). AAMs differentiated in the presence of IL-6 displayed increased arginase activity (D, n = 6) and reduced LPS-evoked nitrate (E) (n = 7) (panels below bar charts are representative immunoblots showing arginase-1 (Arg1) and iNOS (representative of 2–3 experiments); data are mean ± SEM; *, #, p<0.05 compared to control macrophages (MØ) and IL-4+IL-13 AAM).

M-CSF-differentiated bone-marrow (BM)-derived macrophages (∼93% F4/80^+^, <0.5% Gr1^+^ (marker of myeloid-derived suppression cells (MDSCs))) exposed to IL-4+IL-13 for 48 h displayed the canonical features of murine AAMs (Figure 1).

IL-6 is often considered pro-inflammatory and so subsequent experiments focused on its putative role in AAM differentiation. Treatment of murine BALB/c BM-derived macrophages with IL-6 alone did not significantly change macrophage phenotype, based on the parameters assessed, compared to untreated controls (Figure 1). However, when applied as a co-treatment with IL-4+IL-13, IL-6 significantly increased the expression of Arg1, Ym1 and RELMα mRNA (Figure 1A). Q-PCR also revealed increased expression of CD206 mRNA. IL-6 did not affect the reduced CD14 expression seen in AAMs (Figure 1B, C). Bioactivity assays showed a corroborating increase in Arg1 activity and protein expression (Figure 1D) and suppression of nitric oxide production in response to LPS (Figure 1E) in IL-6 co-treated AAMs although the levels of iNOS protein were only slightly reduced in AAM(IL-6) (Figure 1E). Using CAMs as a comparator group, LPS-stimulation of these cells resulted in levels of nitrite similar to those produced by AAMs, corroborating recent findings by other investigators [21]–[23].

**Figure 2:**
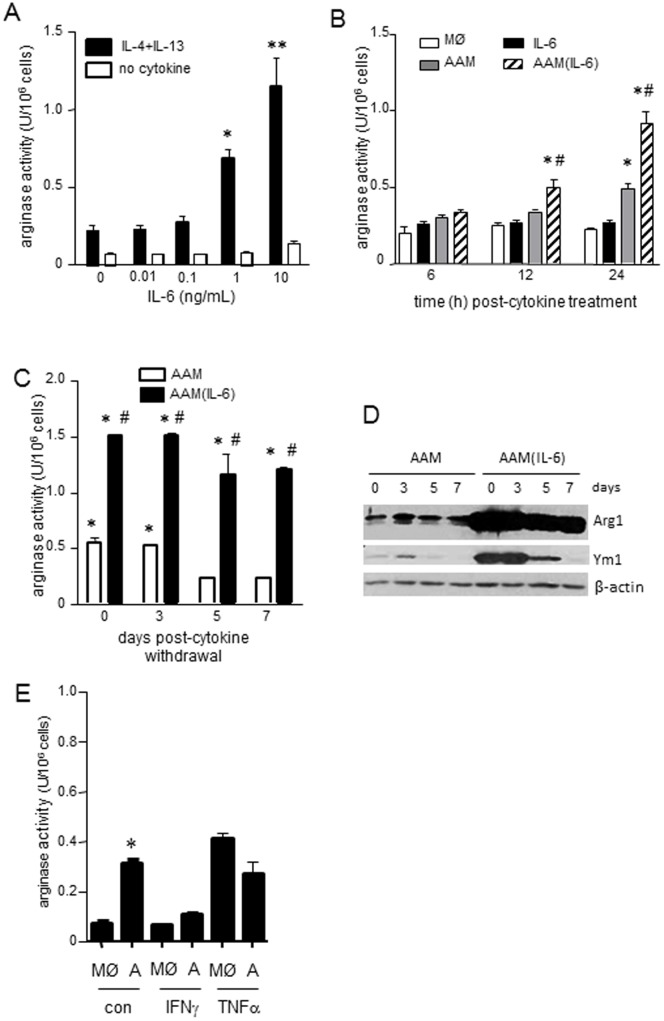
IL-6 alters the polarization of AAMs in a dose- and time-dependent manner. (A) At least 1 ng/mL of IL-6 (48 h) is required to amplify an AAM phenotype. (B) Increased arginase activity is apparent in AAMs (IL-4+IL-13 treated) 24 h post-cytokine treatment, whereas AAM differentiated in the presence of IL-6 (AAM(IL-6)) shows increased arginase activity after 12 h that is enhanced further at 24 h post-cytokine (n = 4), and expression of markers indicative of a murine AAM phenotype is prolonged in AAM(IL-6) compared to time-matched AAMs (C & D, n = 3-4). (E) IFNγ or TNFα (both 10 ng/mL) treatment does not enhance AAM polarization in the same way that IL-6 does (n = 3) (data are mean ± SEM; *, #, **, p<0.05 compared to MØ, AAMs and 0–0.1 ng/mL IL-6 respectively; U, units).

Figures 2A–D presents the time- and dose-dependency of IL-6 synergy with IL-4+IL-13 in the enhancement of an AAM phenotype. A threshold dose of 1 ng/mL was required to observe the effect of IL-6 and at a concentration of 10 ng/mL, enhanced arginase was found 12 h post-treatment. In the presence of IL-6, the AAM phenotype was sustained for 7 days post-withdrawal of the cytokine (last time-point examined).

In order to test the ability of other cytokines typically considered pro-inflammatory on macrophage polarization, AAMs were differentiated in the presence of either IFNγ or TNFα. Co-treatment of macrophages with IL-4+IL-13 + TNFα or IFNγ had no effect on arginase activity compared to IL-4+13 alone (Figure 2E).

### IL-6 differentially regulates cytokine and chemokine output from AAMs and CAMs

**Figure 3:**
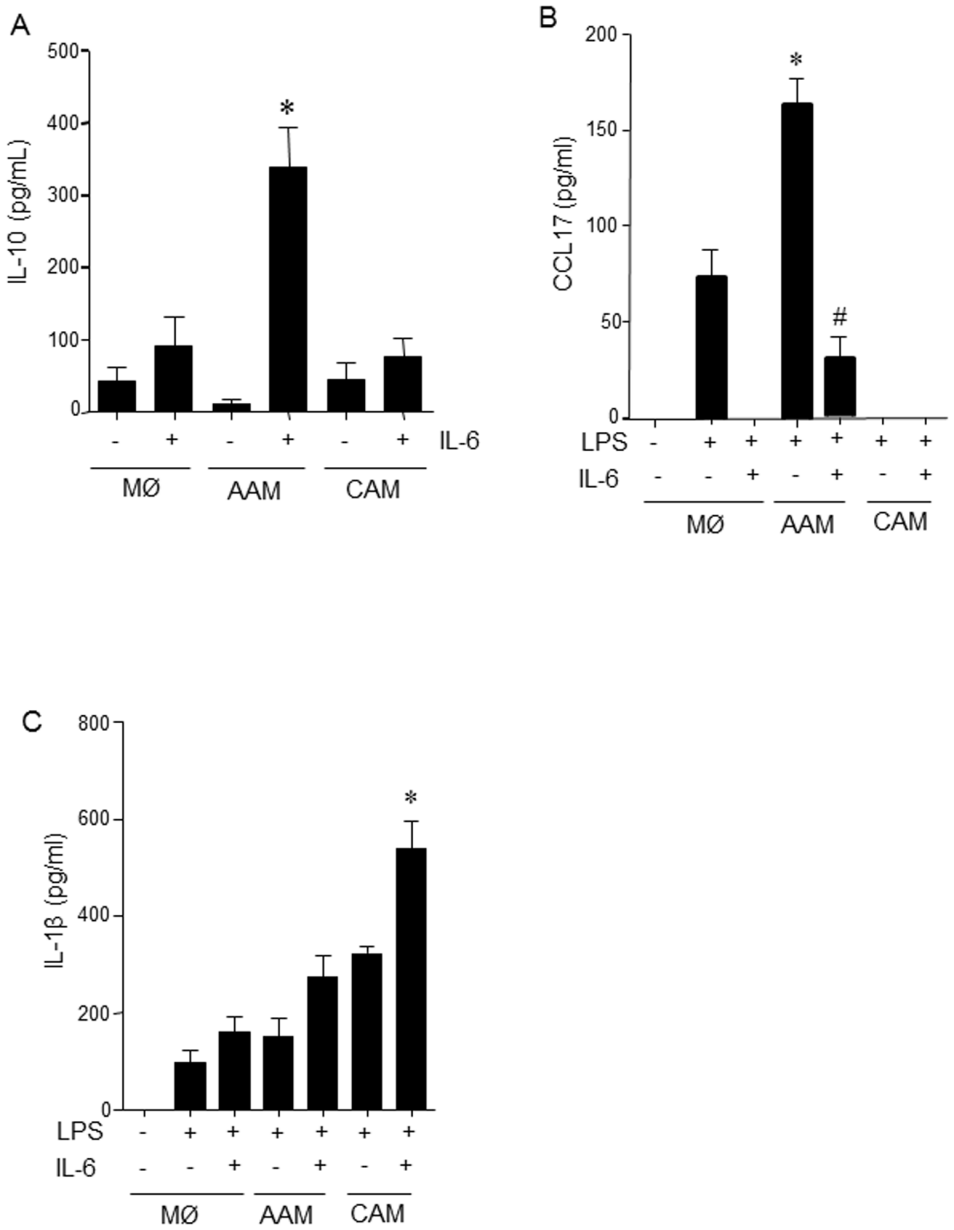
IL-6 alters the cytokine profile of AAM and CAM. Addition of IL-6 (10 ng/mL) to the cytokine milieu during the differentiation of alternatively activated macrophages (AAMs; IL-4+IL-13, 20 ng/ml, 48 h) lead to spontaneous production of IL-10 (A) but suppressed LPS (1 µg/ml, 24 h) evoked production of the AAM chemokine, CCL17 (B). Classically activated macrophages (CAM: IFNγ, 10 ng/mL, 48 h) co-treated with IL-6 produced more IL-β (C) in response to LPS (mean±SEM; n = 3; *p<0.05 compared to control (LPS only), #p<0.05 compared to AAM).

After 48 h in culture, AAM(IL-6) spontaneously produced significant amounts of IL-10 compared to macrophages treated with IL-6 only, AAMs, CAMs or CAM(IL-6) (Figure 3A). Furthermore, LPS-evoked production of the AAM chemokine CCL17 was significantly reduced in AAMs differentiated in the presence of IL-6 (Figure 3B). LPS stimulation evoked IL-1β production by all macrophage subtypes, with IL-6 synergising with CAMs to produce substantially more cytokine (Figure 3C). A small, but statistically significant increase in TNFα was observed in LPS-stimulated CAM(IL-6 compared to CAMs (data not shown). Also, LPS-activated AAM(IL-6) produced less VEGF than AAMs (7±7 vs. 50±17 pg/mL, p<0.05, n = 3), whereas CAM VEGF production was unaffected by IL-6 (CAM  =  158±17; CAM(IL-6) = 174±36 pg/mL, n = 3). Untreated macrophages were not a significant source of VEGF in response to LPS.

### IL-6 enhances IL-4+IL-13 polarization of human blood monocytes into AAMs

**Figure 4:**
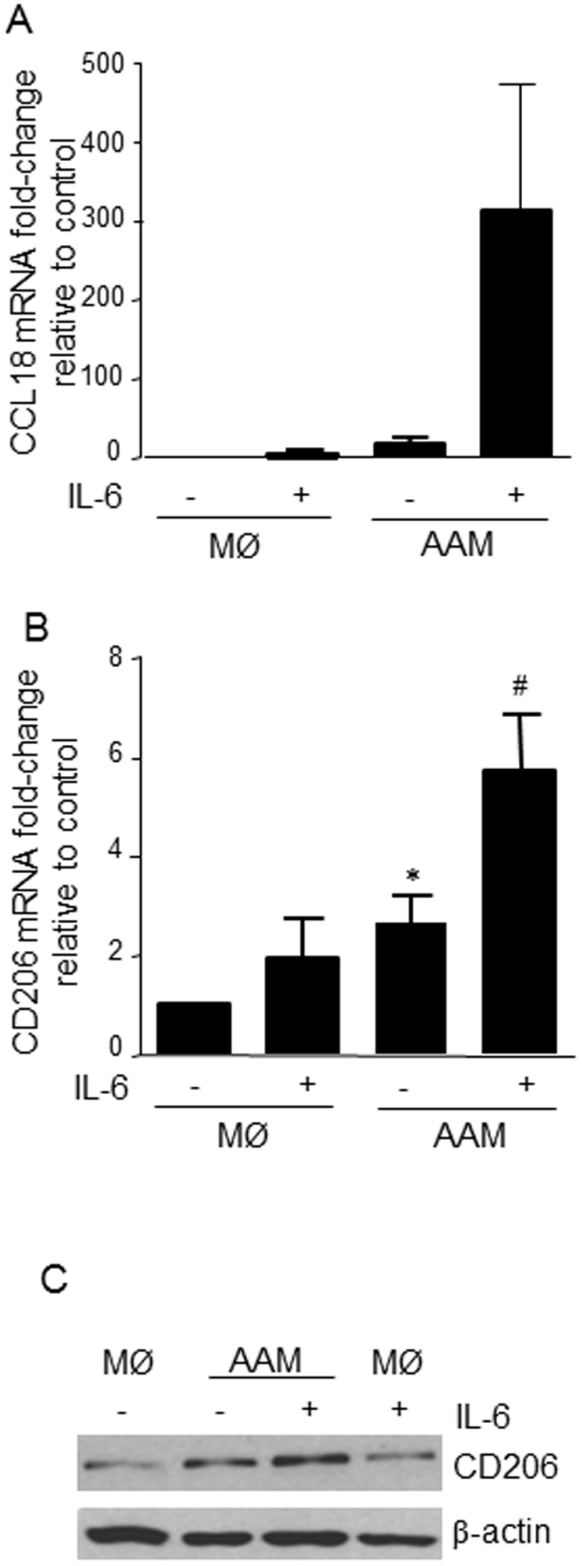
IL-6 enhances expression of markers typical of human AAMs. Stimulation of human AAMs (IL-4+IL-13, 10 ng/mL 48 h) concomitantly with IL-6 (10 ng/mL) enhanced mRNA expression of CCL18 (A) and CD206 (B), and protein expression of CD206 (C) (mean±SEM; n = 7; *p<0.05 compared to macrophages (MØ), ANOVA followed by Dunnetts test; immunoblot is representative of 3 experiments).

IL-6 co-treatment enhanced the expression of CCL18 and CD206 mRNA expression (Figure 4A–B), and CD206 protein expression (Figure 4C) in AAMs differentiated from the blood of healthy volunteers with IL-4+IL-13. Expression of mRNA of the LPS co-receptor, CD14, was not significantly different when human AAMs and AAM(IL-6) were compared (data not shown).

### IL-6-STAT3 signalling is required for an enhanced AAM phenotype

Investigating the mechanism of action of IL-6, the possibility that IL-6 was simply promoting the development of macrophages from precursor cells (i.e. providing more target cells for IL-4+IL-13) as IL-6 has been shown to skew monocytes differentiation towards macrophages and away from dendritic cells was considered [24]. This was not the case, however – although murine peritoneal macrophages had increased constitutive expression of Arg1 activity compared to BMDM, this was still significantly increased by IL-4+IL-13 treatment (48 h) and further enhanced by IL-6 (data not shown).

**Figure 5:**
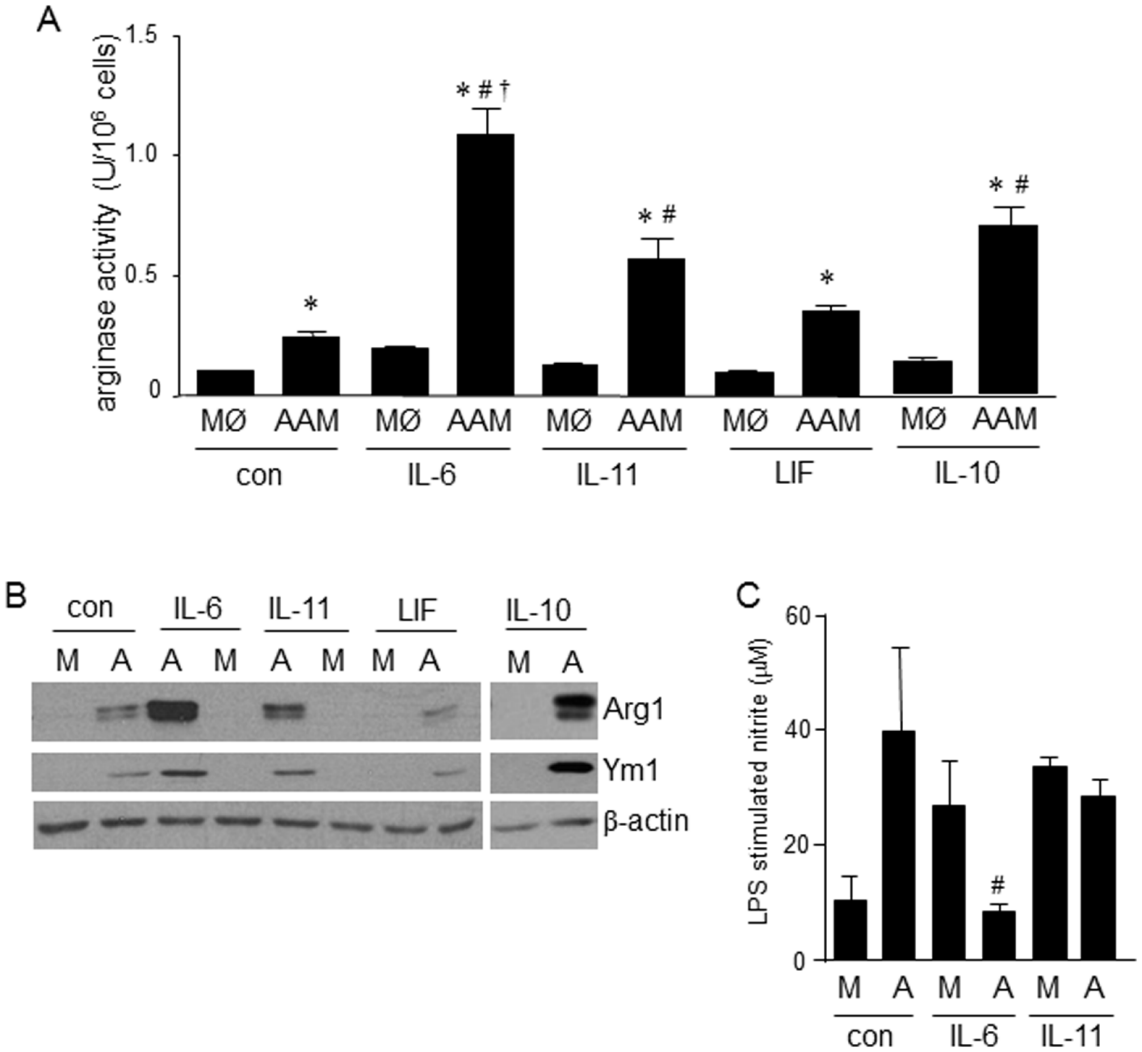
Other cytokines of the IL-6 family are capable of increasing Arg1 and Ym1 expression. (A) Differentiation of bone-marrow derived AAMs (IL-4+IL-13, 20 ng/mL 48 h) in the presence of IL-6, the related family member IL-11 (but not leukemia inhibitory factor (LIF)), and IL-10 (all 10 ng/ml), increased arginase expression (n = 6-7). (B) The degree of arginase expression correlated with the induction of arginase 1 (Arg1) and Ym1 protein expression (n = 3) and (C) of the gp130 family members only IL-6 reduced LPS (1 µg/mL)-stimulated nitrite production by AAMs (mean±SEM; n = 3; A, alternatively activated macrophage; MØ or macrophage; *, #, † p<0.05 compared to macrophages, AAM, and IL-11 or IL-10, respectively).

**Figure 6:**
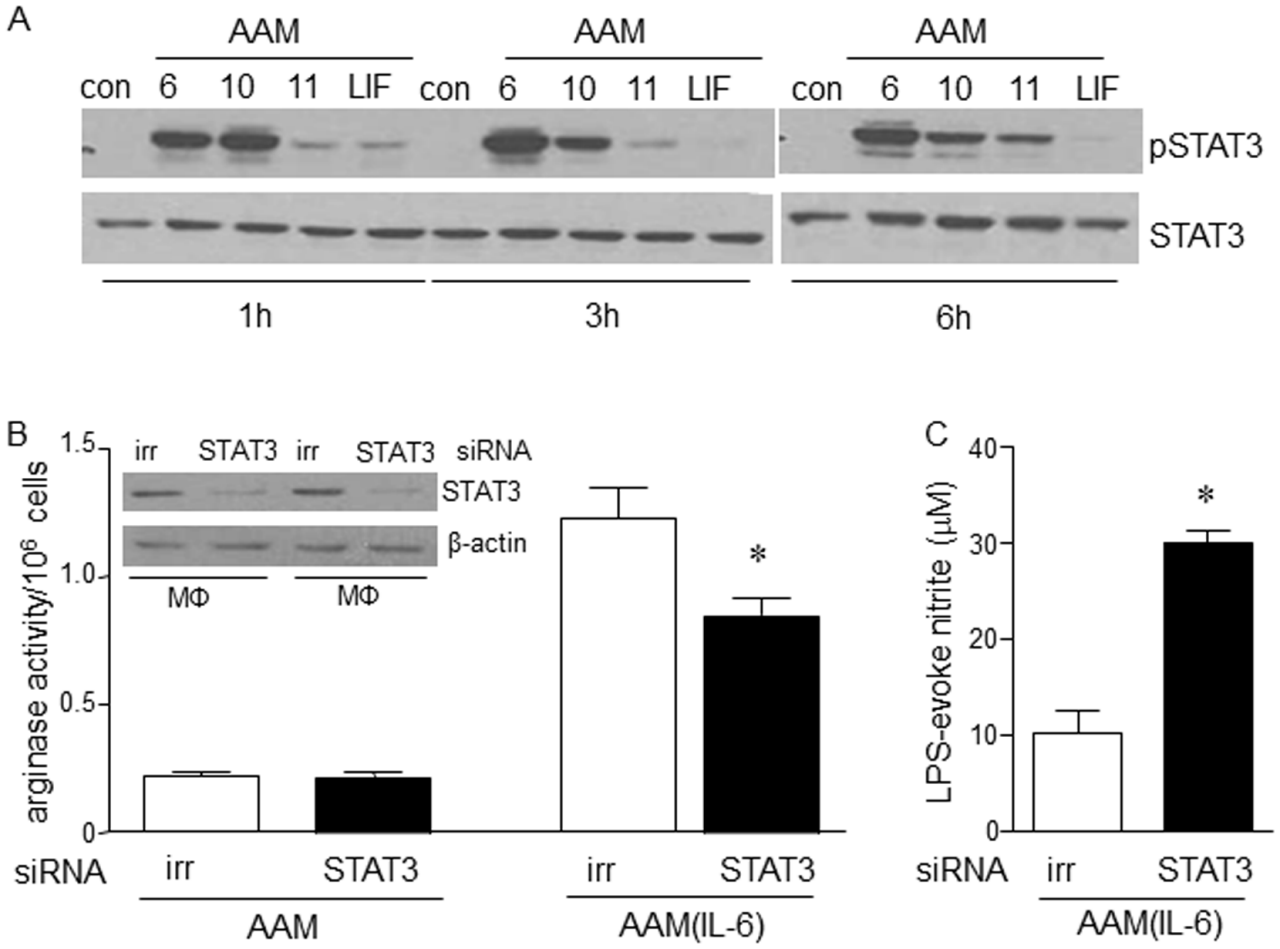
IL-6 induced enhancement of AAM polarization is STAT3-dependent. Panel (A) shows the time-dependent activation of signal transducer and activator of transcription (STAT)-3 as assessed by phosphorylation on immunoblot for IL-6, -10, -11 and LIF (representative of two experiments). siRNA knock-down of STAT3 (insert, panel B) supports the role of STAT3 in the enhanced expression of arginase (B) and reduced LPS-evoked nitrite (C) from AAMs co-treated with IL-6 (AAM(IL-6)) (data are mean±SEM; n = 4; *p<0.05 compared to compared to negative control siRNA treated AAMs).

Assessment of other IL-6 family members that use the gp130 receptor chain, revealed that co-treatment with IL-11, but not leukemia inhibitory factor (LIF), enhanced AAM arginase activity (Fig. 5A) and protein expression of Arg1 and Ym1 (Figure 5B); however, unlike IL-6, IL-11 failed to significantly suppress LPS-stimulated NO production (Figure 5C). We focused on the involvement of STAT3 in the up-regulation of Arg1 and Ym1, as other activators of this transcription factor (IL-10 (Figure 5) and IL-21) can enhance AAM polarization [9] and because there was a strong correlation between levels of phospho-STAT3 and Arg1/Ym1 expression (Figure 6A). siRNA experiments revealed that knock-down of STAT3 (Figure 6B, inset) significantly inhibited the enhancement of arginase activity in AAM(IL-6) (Figure 6B) and also inhibited the suppression of LPS-stimulated nitrite production (Figure 6C).

### IL-6 enhancement of the AAM phenotype is not via IL-10 production

**Figure 7:**
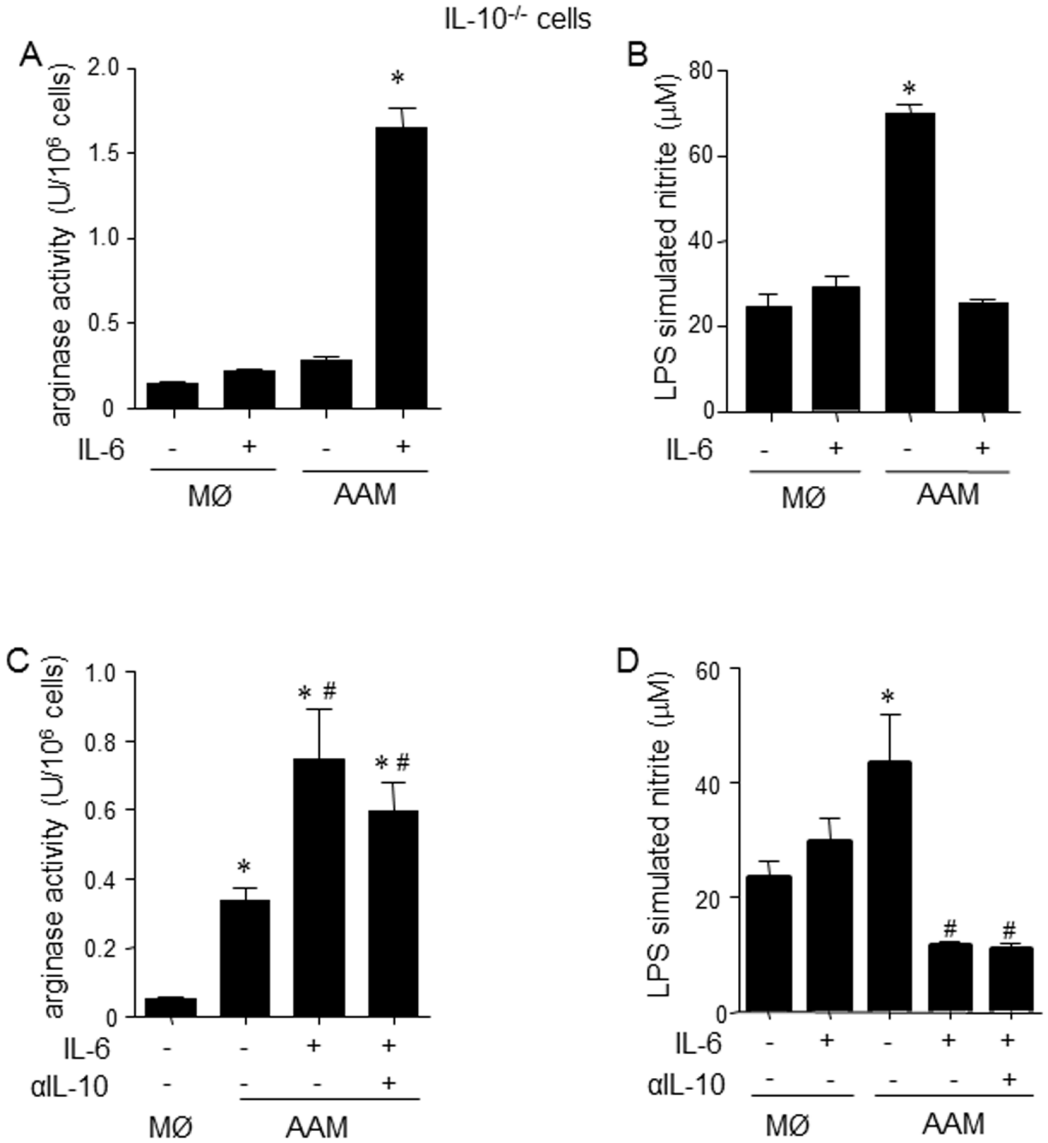
Enhancement of AAM polarization by IL-6 is not dependent on autocrine IL-10. (A–B) Macrophages derived from bone marrow of IL-10^-/-^ mice are still able to enhance arginase activity and suppress nitric oxide in response to IL-4+IL-13 + IL-6 stimulation (*p<0.05 compared to all groups, n = 3). (C–D) Addition of IL-10 neutralizing antibody (10 µg/ml) to the culture medium affected neither the enhanced arginase activity (C) nor the suppressed LPS (1 µg/ml) stimulated nitrite output (D) from wild-type murine bone-marrow derived AAMs differentiated in the presence of IL-6 (n = 3) (mean±SEM, * and # p<0.05 compared to macrophage (MØ) and AAM, respectively).

IL-10 is a target gene of STAT3 and the spontaneous production of IL-10 (Figure 3A) by AAM(IL-6) and the increase in Arg1 and Ym1 observed in macrophages stimulated with IL-4+IL-13 + IL-10 raised the possibility that autocrine IL-10 was mediating the enhanced AAM phenotype. Use of BMDM from IL-10^-/-^ mice showed that this was not the case, as IL-6, in the presence of IL-4+IL-13, was still able to enhance arginase activity and suppress LPS elicited nitric oxide production (Figure 7A and B). These results were corroborated via treatment with a neutralizing antibody against IL-10 (Figure 7C and D).

### Enhancement of the AAM phenotype by IL-6 is partially dependent on up-regulation of the IL-4Rα chain

Signalling through the IL-4 receptor alpha chain is a prerequisite for AAM differentiation [25]. Furthermore, the IL-4Rα chain is a target gene of STAT3 and enhancement of this receptor has been implicated as the mechanism behind other cytokines’ ability to enhance AAM polarization [2], [9], [26].

**Figure 8:**
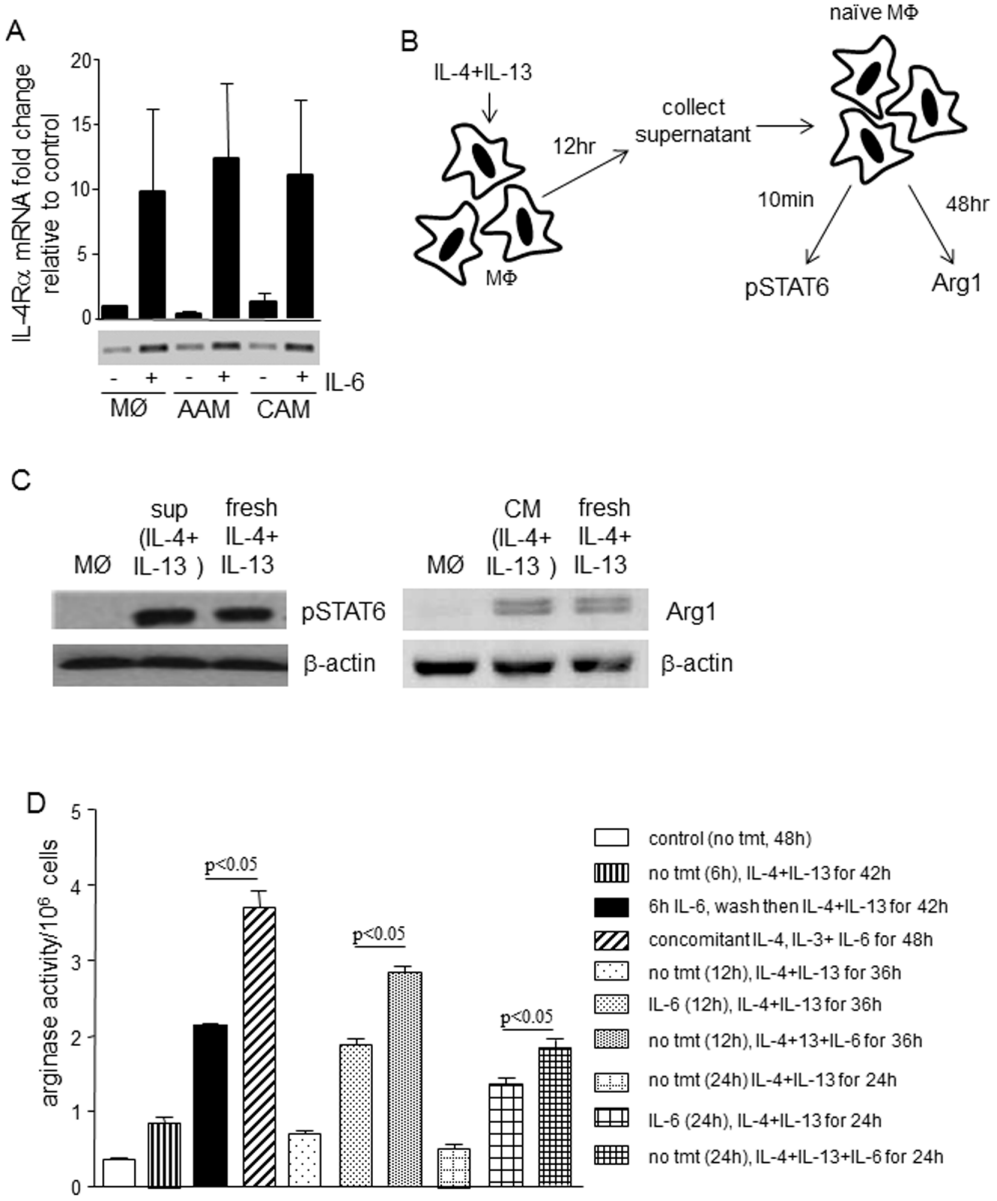
Enhancement of AAM polarization is partly dependent on up-regulation of the IL-4Rα chain. (A) Six hours after IL-6 (10 ng/ml) treatment all sub-classes of macrophage show increased IL-4Rα mRNA expression (n = 3: in gel PCR bands are shown below Q-PCR data for the various conditions). (B) Diagrammatic representation of experimental set-up. (C) Phosphorylated STAT6 levels were equivalent in macrophages stimulated with 12 h conditioned medium supernatants and fresh IL-4+IL-13 for 10 min. Arg1 levels were also equivalent in supernatant versus fresh cytokine treatment as assessed by immunoblotting (n = 2-3). (D) In all instances simultaneous treatment of macrophages with IL-4+IL-13+IL-6 results in enhanced arginase activity compared to time-matched cells receiving an IL-6 pre-treatment followed by a wash and then IL-4+IL-13 (20 ng/mL) exposure (mean±SEM; n = 4).

IL-6 increases expression of IL-4Rα mRNA, as early as 6 h post-IL-6 or IL-4+13 + IL-6 treatment (Figure 8A). Up-regulation of the IL-4 receptor 6 h post-stimulation however, is relevant only if IL-4+IL-13 initially added is still biologically active at this time point. To test this, supernatants were removed 12 h post-cytokine treatment and added onto naïve macrophages cultures (Figure 8B). STAT6 phosphorylation, as an indicator of activation via IL-4Rα, was equivalent in macrophages treated with 12 h supernatant and fresh IL-4+IL-13 (10 min), as was Arg1 protein expression 48 h post-treatment (Figure 8C). However, pre-treatment with IL-6 (which is sufficient to induce IL-4Rα expression) for 6 h or 12 h followed by IL-4+IL-13 for 42 h and 36 h, respectively, did not mimic the effects of IL-4+IL-13 + IL-6 co-treatment (Figure 8D). Simultaneous treatment with IL-4+IL-13+IL-6 consistently evoked the greatest increase in arginase activity, suggesting that increased expression of the IL-4Rα chain is only partly responsible for the IL-6 enhancement of an AAM phenotype.

### AAM(IL-6) have an enhanced ability to suppress T cell cytokine production

**Figure 9:**
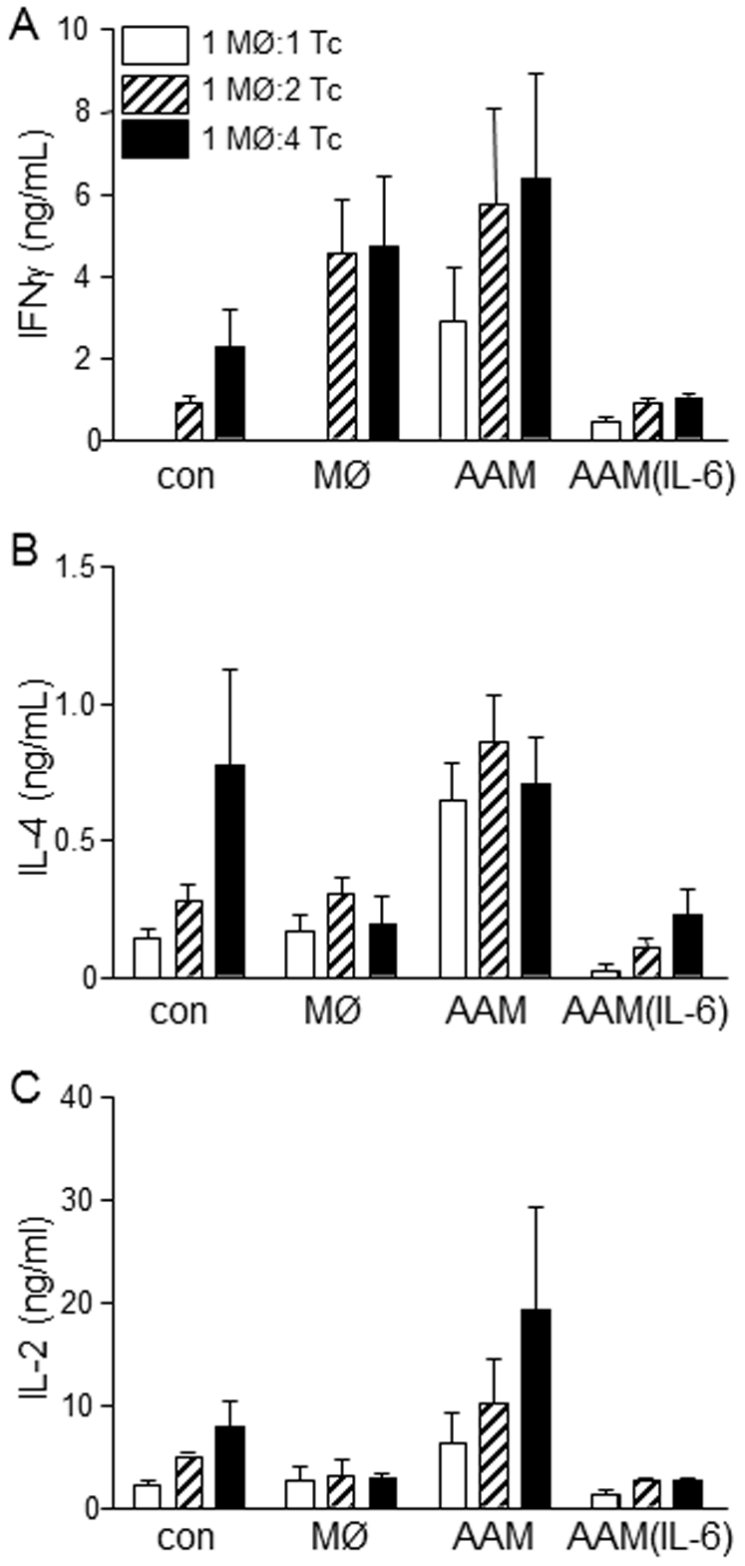
IL-6 treated AAMs display a greater capacity to inhibit T cell cytokine production. Production of IFNγ, IL-4 and IL-2 by anti-CD3+anti-CD28 activated (96 h) murine splenic CD4^+^ T cells is suppressed by co-culture with AAM(IL-6) (i.e. IL-4+IL-13 (20 ng/mL) + IL-6 (10 ng/mL simultaneous application, 48 h exposure) (mean±SEM; n = 4; MØ, macrophage (5×10^4^), Tc, CD4^+^ T cell).

It has been reported that T cell proliferation is suppressed by AAMs. When splenic CD4^+^ T cell were activated by anti-CD3+anti-CD28 antibodies, production of IFNγ, IL-4 and IL-2 was suppressed by co-culture with AAM(IL-6) but not AAMs differentiated with IL-4+IL-13 only (Figure 9).

## Discussion

The plasticity of macrophages, even following differentiation into specific subsets, allows them to adapt to a changing environment to fulfill key immune roles. Here, we opted to focus on IL-6, a cytokine that can directly affect macrophages [24], exert a variety of pro-inflammatory effects [27], and which is produced along with IL-4+IL-13 following infection with helminth parasites [28]. Contrary to our expectation, IL-6 actually reinforced the IL-4+IL-13 polarization of macrophages into AAMs and imparted upon the cells a range of additional immunoregulatory abilities.

IL-6 is a ubiquitously expressed cytokine that, under normal conditions, has many homeostatic functions [29]–[31]. However, it is up-regulated in numerous inflammatory diseases where the majority of findings attest to its pro-inflammatory capacity [13], [32]. For instance, IL-6 can promote the differentiation of pro-inflammatory Th17 cells while suppressing the production of FoxP3^+^ regulatory T cells (Treg) [33]. Given the similarities between the Th17-Treg and CAM-AAM paradigms, we assessed the possibility that IL-6 inhibits differentiation of AAMs, in the same way it does Treg development. However, contrary to this hypothesis, concomitant IL-4+IL-13+IL-6 treatment significantly enhanced and sustained the expression of AAM markers in murine and human cells, compared to AAMs differentiated with IL-4+IL-13 only. Interleukin-6 alone had negligible effects on the expression of Ym1, Relmα and arginase-1, indicating that IL-6 acts to reinforce not trigger an AAM phenotype. The lack of arginase-1 induction by IL-6 contrasts with data from Qualls *et al.,* and this discrepancy may be due differences in the doses of IL-6 used or the presence of IL-10 and GM-CSF (both can stimulate Arg1 expression) in the conditioned medium used in that study [34]. Together, these studies highlight the complexity of control of macrophage phenotype and the role that the microenvironment has in determining macrophage fate.

Functional studies revealed that IL-6 conferred additional immunosuppressive bioactivities on the AAMs. Only the IL-6 co-treated AAMs displayed: (a) spontaneous IL-10 production; (b) suppression of LPS-stimulated nitric oxide production (as assessed by the stable break-down product, nitrite); c) reduced VEGF production; and, (d) inhibition of Th1 and Th2 cytokine production from polyclonally stimulated T cell cultures. Interestingly, IL-6 co-treated AAMs displayed reduced levels of the AAM associated chemokine CCL17 further demonstrating the differential role of IL-6 in macrophage development. This is also important, given that elevated levels of CCL17 are associated with airway inflammation, a process in which AAMs are often considered detrimental [9]. Direct comparisons of CAMs and AAMs suggest that AAMs are not a significant source of NO, but LPS (a stimulus for inducible NO synthase (iNOS) expression) is often used to promote the CAM phenotype [3]. The ability of LPS-challenged AAMs to synthesize NO to a degree similar to that of LPS-challenged CAMs (differentiated with IFNγ) shown here is consistent with the macrophages mandate to phagocytose and kill bacteria, and consistent with findings in other AAM studies [21]–[23]. Indeed, this underscores the novelty of the finding that IL-6 suppresses AAM production of NO in response to LPS, which could be through suppression of iNOS expression (Figure 1E), competition between Arg1 and iNOS for L-arginine, or regulation/modification of iNOS activity, perhaps via alterations in transcription co-factor availability [35].

IL-10 production is often cited as a hallmark of AAMs, and they do generate IL-10 in response to LPS (however, this is also observed in CAMs). However we, and others [9], are unable to detect non-stimulated IL-10 production by IL-4+IL-13 differentiated AAMs. We speculate that the IL-10-AAM literature may be complicated by generic statements applied to a group of cells composed of a number of different macrophage phenotypes [1], [3]. Indeed, this adds to the potential significance of the data herein demonstrating that AAM(IL-6) spontaneously produce IL-10, and this could be important for maintaining the presence and immunoregulatory activity of AAMs that develop in a Th2 (i.e. IL-4+IL-13) dominated environment.

Co-culture of AAM(IL-6) macrophages with activated T cells led to a suppression of the T cell growth associated cytokine, IL-2, as well as the Th1 and Th2 cytokines, IFNγ and IL-4. Interestingly, AAMs themselves were unable to induce a similar or proportional effect, suggesting that although it has been reported that they can suppress proliferation of CD4^+^ T cells [20], cytokine production is not impacted. The mechanism by which IL-6-induced AAMs were able to inhibit this cytokine production was not assessed. There are a number of mechanisms by which T cell inhibition may be occurring, including L-arginine depletion, nitric oxide production, expression of PD-L2 and anti-inflammatory cytokine secretion (IL-10 and TGFβ) [20], [36]. Given the elevated levels of IL-10 and the increased expression of arginase-1 observed by AAM(IL-6) it is feasible that either of these may be responsible for the suppression of T cell activity observed. Production of nitric oxide is an unlikely contributor to the T cell response in this system, as AAM(IL-6) macrophages do not produce observable levels of nitric oxide, even after 96 h co-culture with T cells. In addition suppression of T cells can occur either through the induction of T cell anergy or through the induction of apoptosis, and therefore, further studies are necessary to clarify the exact mechanism by which suppression is occurring.

Despite a focus on its pro-inflammatory nature, IL-6 was shown to promote wound healing in response to chemically-induced burns and suppress inflammation in murine models of alveolar endotoxemia and muscular dystrophy [29], [31], [37]. Substantiating those studies, it has also been shown that IL-6 is necessary for the resolution of inflammation, and its absence prevents proper recovery [29]. We speculate that these beneficial actions could be due, at least in part, by IL-6 induction of an AAM with the immunoregulatory properties described above.

Mechanistic studies revealed that the AAM(IL-6) effect was largely dependent on the canonical transcription factor STAT3, since its depletion in macrophages by siRNA resulted in reduced induction of arginase activity and LPS-stimulation of nitrite production was no longer suppressed (compare Figures 1E and 6C). Corroborating these findings, a qualitative assessment of STAT3 phosphorylation (indicative of activation) by immunoblotting revealed a time-dependent activation of STAT3 by IL-6, IL-10 and IL-11 that mirrored the respective cytokines’ ability to up-regulate Arg1 and Ym1 protein expression. STAT3 has many target genes capable of mediating the IL-6 enhancement of an immunosuppressive AAM phenotype – we focused on spontaneous IL-10 production and expression of the IL-4Rα chain, as other enhancers of AAM polarization operate via these mechanisms [2], [9], [26].

Use of IL-10 neutralizing antibodies and BMDM from IL-10^-/-^ mice revealed that the IL-6 effect on AAMs was not due to a trans-activation event via autocrine IL-10. Interleukin-4 receptor signaling is an absolute requirement for the AAMs assessed here (confirmed using BMDM from IL-4Rα^-/-^ mice, pers. obs). The increased IL-4Rα chain expression combined with bioavailability of IL-4+IL-13 (indicated by induction of phospho-STAT6 in naïve cells treated with supernatants from AAM cultures) could account for much of the enhanced expression of Ym1, Relmα and Arg1 in the AAM(IL-6). However, detailed kinetic analyses revealed that IL-6 was most effective in promoting an AAM phenotype when added simultaneously with IL-4+IL-13, suggesting that the IL-6 effect goes beyond induction of the IL-4 receptor. Moreover, the immunoregulatory functions of the AAM(IL-6) attest to other, yet to be defined, mechanisms driven either exclusively by IL-6 receptor ligation (and STAT3 signaling) or interaction and synergistic communication between the IL-4/IL-13 and IL-6 pathways.

LPS-evoked pro-inflammatory cytokine output was increased in IL-6+IFNγ differentiated macrophages compared to IFNγ only treated cells (i.e. CAMs), underscoring the ability of IL-6 to affect macrophage biology in general [38]. Thus, IL-6 may be a key accessory cytokine that promotes the development of a phenotype to which the macrophage has been committed via simultaneous exposure to IL-4±IL-13 or IFNγ. That is, IL-6 is important in promoting and sustaining a macrophage phenotype determined by the microenvironment, setting the stage for the cell to be pro-resolution or pro-inflammatory. These processes are important in wound healing and combating infection.

In conclusion, the data herein are compatible with a scenario in which the ubiquitous IL-6 can enhance both AAM and CAM phenotypes. The AAM is not only sustained, it displays additional immunoregulatory functions not apparent in the parent AAM, suggesting that the AAM(IL-6) may be a ‘unique’ regulatory cell. Finally, the novel finding of a functionally distinct, and putatively anti-inflammatory AAM(IL-6) may be an important element to consider in the development of therapies based on inhibition of IL-6.
